# Acute Detubulation of Ventricular Myocytes Amplifies the Inhibitory Effect of Cholinergic Agonist on Intracellular Ca^2+^ Transients

**DOI:** 10.3389/fphys.2021.725798

**Published:** 2021-08-26

**Authors:** Andriy E. Belevych, Vladimir Bogdanov, Dmitry A. Terentyev, Sandor Gyorke

**Affiliations:** ^1^Department of Physiology and Cell Biology, The Ohio State University, Columbus, OH, United States; ^2^Davis Heart and Lung Research Institute, The Ohio State University Wexner Medical Center, Columbus, OH, United States

**Keywords:** excitation-contraction coupling, t-tubule, muscarinic receptor agonist, calcium microdomains, ventricular myocyte

## Abstract

Muscarinic receptors expressed in cardiac myocytes play a critical role in the regulation of heart function by the parasympathetic nervous system. How the structural organization of cardiac myocytes affects the regulation of Ca^2+^ handling by muscarinic receptors is not well-defined. Using confocal Ca^2+^ imaging, patch-clamp techniques, and immunocytochemistry, the relationship between t-tubule density and cholinergic regulation of intracellular Ca^2+^ in normal murine ventricular myocytes and myocytes with acute disruption of the t-tubule system caused by formamide treatment was studied. The inhibitory effect of muscarinic receptor agonist carbachol (CCh, 10 μM) on the amplitude of Ca^2+^ transients, evoked by field-stimulation in the presence of 100 nM isoproterenol (Iso), a β-adrenergic agonist, was directly proportional to the level of myocyte detubulation. The timing of the maximal rate of fluorescence increase of fluo-4, a Ca^2+^-sensitive dye, was used to classify image pixels into the regions functionally coupled or uncoupled to the sarcolemmal Ca^2+^ influx (I_Ca_). CCh decreased the fraction of coupled regions and suppressed Ca^2+^ propagation from sarcolemma inside the cell. Formamide treatment reduced I_Ca_ density and decreased sarcoplasmic reticulum (SR) Ca^2+^ content. CCh did not change SR Ca^2+^ content in Iso-stimulated control and formamide-treated myocytes. CCh inhibited peak I_Ca_ recorded in the presence of Iso by ∼20% in both the control and detubulated myocytes. Reducing I_Ca_ amplitude up to 40% by changing the voltage step levels from 0 to –25 mV decreased Ca^2+^ transients in formamide-treated but not in control myocytes in the presence of Iso. CCh inhibited CaMKII activity, whereas CaMKII inhibition with KN93 mimicked the effect of CCh on Ca^2+^ transients in formamide-treated myocytes. It was concluded that the downregulation of t-tubules coupled with the diminished efficiency of excitation–contraction coupling, increases the sensitivity of Ca^2+^ release and propagation to muscarinic receptor-mediated inhibition of both I_Ca_ and CaMKII activity.

## Introduction

The autonomic nervous system exerts its effects on the mechanical and electrical activity of the heart in part *via* β-adrenergic and muscarinic receptors expressed in ventricular myocytes. β-Adrenergic receptors mediate stimulatory effects of the sympathetic nervous system *via* cAMP-dependent activation of Ca^2+^ handling proteins, such as the L-type Ca^2+^ channels, sarcoplasmic reticulum (SR) Ca^2+^ ATPase, and RyR2, SR Ca^2+^ release channels (Bers, 2001). Muscarinic receptors contribute to the inhibitory effects of the parasympathetic system mostly by antagonizing β-adrenergic responses (Löffelholz and Pappano, 1985; Harvey and Belevych, 2003). In cardiac diseases, such as heart failure, the abnormal autonomic regulation of cardiac function is usually accompanied by the structural remodeling of the heart that at the cellular level is often associated with downregulation of the t-tubule system (Guo et al., 2013; Poláková and Sobie, 2013). T-tubules, branched invaginations of sarcolemma occurring at the Z-lines in ventricular myocytes, provide critical support for excitation–contraction coupling (ECC) by aligning the sarcolemmal Ca^2+^ influx channels (L-type) with the SR Ca^2+^ release channels (RyR2s) (Brette and Orchard, 2003). The juxtaposition of t-tubule and SR membranes creates a unique dyadic microdomain, characterized by high Ca^2+^ fluxes and distinct patterns of Ca^2+^-dependent signaling (Bers, 2001; Brette and Orchard, 2003; Jones et al., 2018). Accordingly, RyR2 coupled to t-tubules and those that are not associated with the dyadic domain (uncoupled) display different functional properties (Biesmans et al., 2011; Dries et al., 2013; Belevych et al., 2017). Growing evidence suggests that β-adrenergic receptors differentially regulate the L-type and RyR2 Ca^2+^ channels in dyadic vs. extra-dyadic compartments (Nikolaev et al., 2010; Dries et al., 2016; Belevych et al., 2017). However, little is known about the role of t-tubules in muscarinic receptor–mediated regulation of intracellular Ca^2+^ handling in ventricular myocytes.

In the present study, the mechanisms underlying the regulation of ECC by muscarinic receptor stimulation in the setting of acute t-tubule disruption were addressed. Using formamide treatment, a well-characterized approach to detubulate ventricular myocytes, it was found that the downregulation of t-tubules amplifies the inhibitory effects of muscarinic receptor stimulation on ECC. Mechanistically, this effect was attributed to the increased sensitivity of ECC to muscarinic receptor-mediated effects, inhibition of the L-type Ca^2+^ channels, and decrease in CaMKII-dependent activity of RyR2.

## Materials and Methods

### Ventricular Myocyte Isolation

All animal procedures were approved by the Ohio State University Institutional Animal Care and Use Committee and conformed to the Guide for the Care and Use of Laboratory Animals published by the US National Institute of Health (NIH Publication No. 85-23, revised 2011). Ventricular myocytes were isolated from 2 to 6-month-old C57BL/6J mice (Jackson Laboratory Stock No: 000664) of either sex. Mice were anesthetized with 5% isoflurane in 95% oxygen, hearts were rapidly excised and cannulated through the aorta for perfusion with an ice-cold calcium-free solution containing (in mM): 140 NaCl, 5.4 KCl, 0.5 MgCl_2_, 10 HEPES, and 5.5 glucose with pH 7.4. Using the Langendorff apparatus, hearts were perfused with the a Ca^2+^-free solution at 37°C for 5 min that was followed by perfusion with a Ca^2+^-free solution containing Liberase TH (0.24U; Roche). Following enzymatic digestion (10–20 min), hearts were minced and triturated in the perfusion solution containing 2% BSA. Following two rounds of gravity sedimentation, ventricular myocytes were plated on the laminin (40 μg/ml) and stored at room temperature (RT). Isolated cells were used for experiments within 6 h after isolation.

### Ca^2+^ Imaging and Patch-Clamp Techniques

The cellular experiments were performed using an open bath imaging chamber (Warner Instruments, CT, United States) that was continuously perfused with an external solution containing the following (in mM): 140 NaCl, 5.4 KCl, 2.0 CaCl_2_, 0.5 MgCl_2_, 5.6 glucose, and 10 HEPES (pH 7.4). Myocytes were incubated in a low Ca^2+^ external solution (0.5 mM CaCl_2_) containing 9 μM Fluo-4 AM (Thermo Fisher Scientific, MA, United States) for 20 min at RT. Following 15–20 min of de-esterification in the dye-free external solution, intracellular Ca^2+^ transients were induced by electrical field stimulation (SD9 stimulator, Grass Technologies/Astro-Med Inc., RI, United States) using a pair of platinum electrodes. The following extracellular solution was used for the field-stimulation experiments (mM): 140 NaCl, 5.4 KCl, 2.0 CaCl_2_, 0.5 MgCl_2_, 10 HEPES, and 5.6 glucose (pH 7.4).

In experiments directed to study the regulation of the L-type Ca^2+^ channels by muscarinic receptor agonist, Ca^2+^ currents were recorded in the extracellular solution containing 1 mM CaCl_2_ and 5.4 mM CsCl replacing KCl. Patch pipettes were filled with a solution that contained in mM: 123 CsCl, 20 TEACl, 5 MgATP, 10 NaCl, 1 MgCl_2_, 0.1 Tris GTP, 5 EGTA, and 10 HEPES (pH 7.2). Whole-cell patch-clamp configuration was established using an Axopatch 200B amplifier coupled to Digidata1440 data acquisition system (Axon Instruments Inc./Molecular Devices, CA, United States). In these experiments, the Ca^2+^ currents were evoked by 100 ms depolarization steps to 0 mV every 10 s. The depolarization steps were preceded by a 100 ms step from –80 to –50 mV to inactivate Na^+^ currents.

Imaging of the intracellular Ca^2+^ transients was performed using Olympus FluoView FV 1000 (Olympus America Inc., PA, United States) confocal microscope system equipped with x60 oil-immersion objective lens (NA 1.4). Fluo-4 was excited with a 488 nm line of argon laser, and the signal was collected at 500–600 nm wavelengths. Linescan images were acquired along the central axis of the myocytes at a speed of 2.1 ms per line. Fluorescence signals were normalized to the baseline cellular fluorescence (F_0_).

To record Ca^2+^ currents simultaneously with the intracellular Ca^2+^ transients, the extracellular solution containing 1 mM CaCl_2_ was used and in the patch pipette solution, 5 mM EGTA was replaced with 0.1 mM fluo-3 K_5_ (ThermoFisher Scientific, MA, United States). In these experiments, the following voltage protocol was used: 100 ms voltage ramp was applied from the holding potential of –80 to –45 mV, at which level the voltage was kept for 500 ms followed by a 50 ms depolarization step to either –25, or –20, or 0 mV to induce the Ca^2+^ currents of different amplitude. The Ca^2+^ currents were evoked every 8 s. A train of 10 pulses from –80 to –10 mV was applied at 2 Hz to maintain steady SR Ca^2+^ loading before each Ca^2+^ current-inducing voltage protocol.

The SR Ca^2+^ content was assessed by the rapid application of an extracellular solution containing 5 mM caffeine and 20 mM 2,3-butanedione monoxime, as previously described (Kashimura et al., 2010). All recordings were made at RT.

Detubulation of the myocytes was induced following 15 min incubation in the low-Ca^2+^ external solution containing 1.5 M formamide (Brette et al., 2002). T-tubules were labeled with 3.3 μg/ml of di4-AN(F)EPPTEA at RT for 5–10 min.

### Immunocytochemistry

Plated on laminin cardiomyocytes were incubated for 10 min in the external solution containing either isoproterenol (Iso, 100 nM), or Iso (100 nM) plus carbachol (CCh) (10 μM), or Iso (100 nM) plus KN93 (2 μM). Myocytes were electrically stimulated for 1 min at 2 Hz and 1 min at 1 Hz. Following stimulation protocol, cells were immediately fixed with 4% paraformaldehyde (10 min at RT) and washed with phosphate-buffered saline (PBS, 3 × 10 min at RT). Fixed myocytes were permeabilized with 0.25% Triton X-100 in PBS (15 min, RT) and incubated in blocking solution (BlockAid^TM^ Blocking Solution, Thermo Fisher Scientific, MA, United States) for 60 min at RT. Following overnight incubation at 4°C in the blocking solution containing primary antibody (1:150 dilution, anti-CaMKII (phospho T286) antibody, ab32678, Abcam, Cambridge, United Kingdom) and several rounds of PBS washes, myocytes were incubated in the blocking solution containing secondary antibody (1:500 dilution, goat anti-rabbit IgG [H + L], Invitrogen) for 90 min at RT. After 3 × 5 min of PBS washes, the samples were mounted using ProLong^TM^ Gold Antifade Mountant (Thermo Fisher Scientific, MA, United States). Images were acquired using an Olympus FluoView FV 1000 (Olympus America Inc., PA, United States) confocal microscope system equipped with a ×60 oil-immersion objective lens (NA 1.4). Following subtraction of the non-cellular background signal, average fluorescence, excluding nuclear area, was calculated for each myocyte and normalized to the value of mean fluorescence value obtained from a group of myocytes incubated in 100 nM Iso.

### Image Analysis

The activation time of Ca^2+^ release during the electrical stimulation was used to assess the proximity to the sarcolemma (Dries et al., 2013, 2016; Belevych et al., 2017). Time to the peak of fluorescence derivative (d*F/*d*t*) during the rising phase of Ca^2+^ transients was calculated for each pixel. The obtained distribution of time to (d*F/*d*t*)_max_ was used to classify pixels into “early” and “delayed,” as described in the “Results” section. T-tubule density was analyzed according to Wagner et al. (2014).

### Statistical Analysis

Images were analyzed using MATLAB (2017b, The MathWorks, Inc., MA, United States) and ImageJ (Rasband, W.S., ImageJ, US National Institutes of Health, Bethesda, Maryland, United States^1^, 1997–2018) software. Aggregate data were analyzed using the R software environment (The R Project for Statistical Computing^2^) and OriginPro 2020b (OriginLab Corp, MA, United States). Results are expressed as the mean ± SEM. Statistical significance was determined using either ANOVA with Tukey’s *post hoc* test or Student’s *t*-test with *p*-values of 0.05. Linear fits to the data were compared using the *F* test.

### Reagents

All materials were obtained from Sigma-Aldrich (St. Louis, MI, United States) unless specified otherwise.

## Results

### Effect of Muscarinic Receptor Stimulation on Intracellular Ca^2+^ Transients in Control and Detubulated Ventricular Myocytes

**FIGURE 1:**
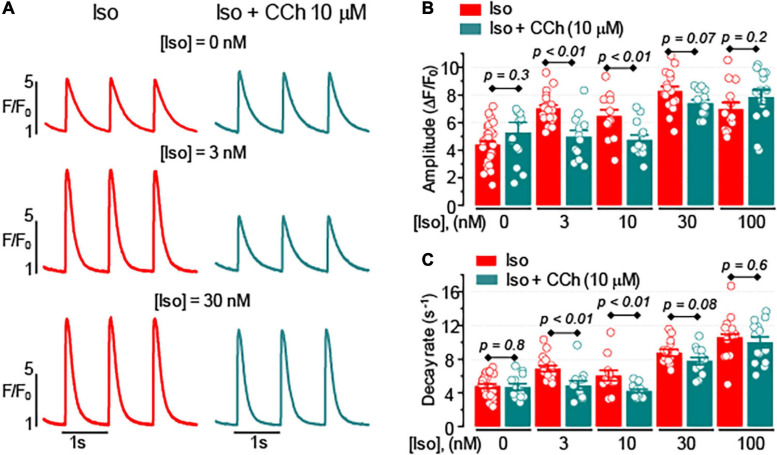
The effect of muscarinic receptor stimulation on cytoplasmic Ca^2+^ transients depends on the level of β-adrenergic receptor stimulation. **(A)** Representative fluorescence profiles obtained from the line-scan images recorded in mouse ventricular myocytes electrically stimulated at 1 Hz in the presence of isoproterenol (Iso) alone or the presence of Iso plus 10 μM carbachol (CCh). Bar graphs with individual data points illustrate the effect of 10 μM CCh on the amplitude **(B)** and the decay rate **(C)** of Ca^2+^ transients recorded in the presence of various concentrations of Iso.

We studied the effects of the muscarinic receptor agonist, CCh, on Ca^2+^ transients evoked by 1 Hz electrical field stimulation in ventricular myocytes isolated from WT mouse heart. In ventricular myocytes, the effect of muscarinic receptor stimulation is generally attributed to the modulation of cAMP-dependent responses coupled to β-adrenergic receptor stimulation (Löffelholz and Pappano, 1985; Endoh, 1999; Harvey and Belevych, 2003). Indeed, in the absence of β-adrenergic receptor stimulation, CCh (10 μM) did not affect the properties of Ca^2+^ transients (Figure 1). However, in the presence of 3–10 nM isoproterenol (Iso), a β-adrenergic receptor agonist, CCh inhibited the amplitude and decay rate of Ca^2+^ transients by 25–30% (Figure 1). The inhibitory effects of CCh on Ca^2+^ transients were not observed in the presence of 30–100 nM Iso in control ventricular myocytes (Figure 1 and Supplementary Table 1).

**FIGURE 2:**
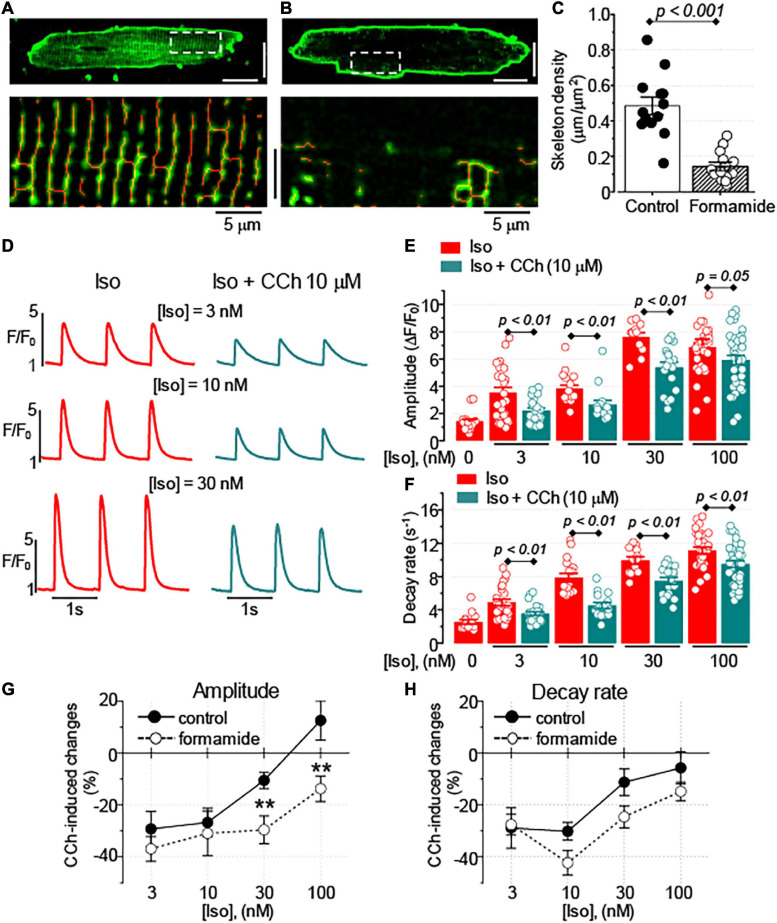
Formamide treatment disrupts t-tubule organization and amplifies the inhibitory effect of CCh. Representative images of control **(A)** and formamide-treated **(B)** ventricular myocytes stained with membrane dye Di-4-AN(F)EPPTEA. Lower panels represent scaled-up regions indicated by white line rectangles and illustrate morphological skeletons of t-tubules (red lines). White scale bars are 20 μm. **(C)** Summary data along with the individual data points are shown for the density of t-tubules in control (*n* = 13) and formamide-treated (*n* = 13) ventricular myocytes, respectively. **(D)** Representative fluorescence profiles were obtained from the line-scan images recorded in formamide-treated ventricular myocytes electrically stimulated at 1 Hz in the presence of Iso alone or the presence of Iso plus 10 μM CCh. Bar graphs with individual data points illustrate the effect of 10 μM CCh on the amplitude **(E)** and the decay rate **(F)** of Ca^2+^ transients recorded in the presence of various concentrations of Iso in formamide-treated myocytes. CCh–induced changes in the amplitude **(G)** and the decay rate **(H)** of Ca^2+^ transients recorded in control and formamide-treated myocytes are plotted against the concentration of Iso. **p < 0.01 (control *vs.* formamide).

In cardiac myocytes, deep invaginations of the sarcolemma, known as t-tubules, have a significant impact on the regulation of intracellular Ca^2+^ handling (Brette and Orchard, 2003). To test whether the regulation of Ca^2+^ handling by muscarinic receptors in ventricular myocytes depends on the spatial organization of t-tubules, the effects of CCh in myocytes with reduced t-tubule density resulting from formamide treatment were studied (Brette et al., 2002). As shown in Figures 2A–C, formamide treatment reduced t-tubules density in the ventricular myocytes by ∼70%. The formamide-treated myocytes, CCh (10 μM) inhibited the amplitude of Ca^2+^ transients in the presence of 3, 10, and 30 nM Iso, and slowed Ca^2+^ transient decay in the presence of Iso at all concentrations studied (Figures 2E,F and Supplementary Table 2). Comparison of CCh-induced changes in Ca^2+^ transients in control vs. formamide-treated myocytes (Figures 2G,H) revealed that t-tubule downregulation was associated with the more pronounced inhibitory effect on CCh on the amplitude of Ca^2+^ transients recorded in the presence of 30 and 100 nM Iso.

### The Relationship Between T-Tubule Density and CCh Effect on Ca^2+^ Transients in Ventricular Myocytes

**FIGURE 3:**
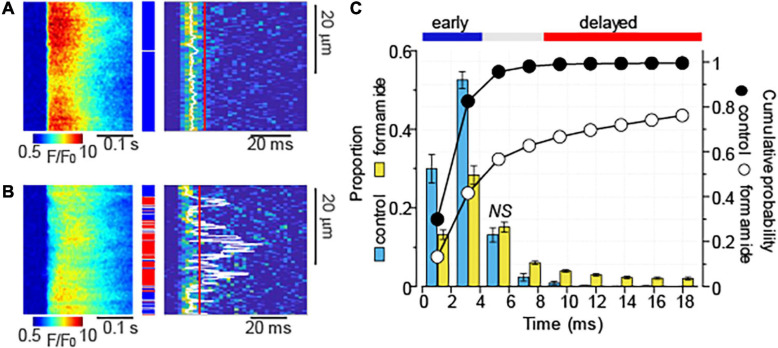
Formamide treatment delays Ca^2+^ release activation. **(A,B)** Linescan images of fluo-4 fluorescence (left panels) and corresponding images illustrating the rate of fluorescence change (right panels) recorded in control **(A)** and formamide-treated **(B)** ventricular myocytes in the presence of Iso (100 nM). Ca^2+^ transients were induced by electrical field stimulation at 1 Hz. White lines indicate the maximal rate of fluorescence increase [(d*F/*d*t*)_max_] that was calculated by averaging data from at least five consecutive Ca^2+^ transients. **(C)** Average time distribution of (d*F/*d*t*)_max_ obtained from the line-scan images of control (*n* = 12) and formamide-treated (*n* = 33) myocytes. Time distribution in each myocyte was adjusted to the timing of fastest (d*F*/d*t*)_max_. The time distribution bin is 2.1 ms. Formamide treatment significantly changed the relative frequency of occurrence of (d*F*/d*t*)_max_ at each time interval except where indicated by NS (not significant). Blue and red lines indicate the time intervals used for the classification of image pixels into the “early” [blue rectangles in panel **(A,B)**] and “delayed” [red rectangles in panel **(A,B)**] regions, respectively.

The time delay between the electrical stimulus and activation of Ca^2+^ release has been previously used to characterize the proximity of Ca^2+^ release units to the sarcolemma (Dries et al., 2013, 2016; Belevych et al., 2017). We recorded Ca^2+^ transients induced by 1 Hz stimulation in the presence of Iso (100 nM) in control and formamide-treated myocytes and obtained time distribution of maximal rate of fluorescence increase (d*F*/d*t*)_max_ for each line-scan pixel in each myocyte (Figure 3). As demonstrated in Figures 3A,C, in control myocytes, Ca^2+^ release was highly synchronous [(d*F*/d*t*)_max_ for 98% of line-scan pixels occurred within 6.3 ms]. Formamide-treatment decreased the fraction of fast response regions (first 4.2 ms) by about 50% and significantly increased the fraction of regions displaying (d*F*/d*t*)_max_ after 6.3 ms (Figures 3B,C). Assuming that the effects of formamide treatment on Ca^2+^ transients stem mostly from decreased t-tubule density (Brette et al., 2002), we classified regions displaying (d*F*/d*t*)_max_ after 8.5 ms as “delayed,” or uncoupled from the t-tubules, whereas line-scan pixels with (d*F*/d*t*)_max_ occurring within first 4.2 ms were classified as “early,” or coupled to t-tubule regions.

**FIGURE 4:**
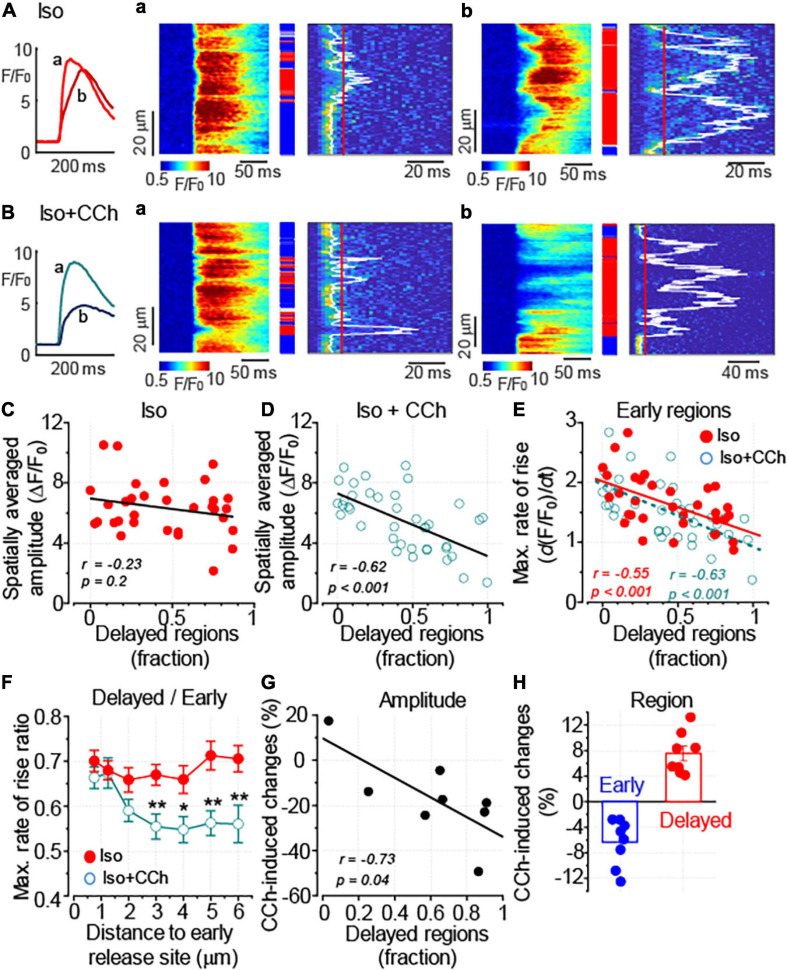
Downregulation of t-tubules augments the anti-adrenergic effect of CCh on the amplitude of Ca^2+^ transients. **(A,B)** Ca^2+^ transients were recorded in formamide-treated ventricular myocyte in the presence of 100 nM Iso alone (**A**, *n* = 32) and the presence of 100 nM Iso plus 10 μM CCh (**B**, *n* = 39). The inset on the left illustrates spatially averaged fluorescence profiles recorded from the cells displaying moderate (a, corresponding to the line-scan image in panel a) and pronounced (b, corresponding to the line-scan image in panel b) levels of detubulation, respectively. **(A**,a,b,**B**,a,b**)** Linescan images of fluo-4 fluorescence (left) and corresponding images illustrating the rate of fluorescence change (right). White lines indicate the timing of (d*F/*d*t*)_max_, and red lines indicate the time point used to differentiate delayed regions (red rectangles). Early and intermediate regions are indicated by blue and gray rectangles, respectively. **(C,D)** Regression analysis of the relationship between the detubulation level and the amplitude of Ca^2+^ transients showed the lack of correlation for the transients recorded in a presence of Iso alone **(C)** and the inverse correlation for the transients recorded in a presence of Iso plus CCh **(D)**. **(E)** The relationships between the detubulation level and (d*F/*d*t*)_max_ at the early Ca^2+^ release regions recorded in the presence of Iso alone and in the presence of Iso plus CCh are shown along with the linear fits to the data. The two datasets were not significantly different (*F* test, *p* = 0.3). **(F)** CCh suppressed propagation of Ca^2+^ signal from the sarcolemma inside the cell. The propagation of Ca^2+^ was characterized by plotting the ratio of (d*F/*d*t*)_max_ recorded at the delayed regions to that measured at the early regions vs. the distance to the nearest early Ca^2+^ release site (*p < 0.05; **p < 0.01). **(G)** CCh-induced changes in the Ca^2+^ transient amplitude plotted against the level of myocyte detubulation. Ca^2+^ transients were recorded in the presence of Iso (100 nM) alone and the presence of Iso (100 nM) plus CCh (10 μM) in the same myocytes. The delayed region fraction used in this graph was calculated from the line-scan images obtained before the application of CCh (in the presence of Iso alone). **(H)** CCh reduces the fraction of early and increases the fraction of delayed regions, respectively. **(C–E,G)** *r*, Pearson’s correlation coefficient; *p*, the probability that the slope of the regression curve is different from 0.

Following formamide treatment, the proportion of the delayed regions was on average 44% in myocytes treated with Iso alone and 42% in cells challenged with Iso plus CCh (*p* = 0.8). Figures 4A,B shows line-scan images of myocytes displaying a low and a high proportion of delayed Ca^2+^ release regions. As illustrated in Figures 4A,C, the proportion of the delayed regions did not significantly affect the amplitude of Ca^2+^ transients observed in the presence of 100 nM Iso. In contrast, peak Ca^2+^ transient recorded in the presence of Iso plus CCh inversely correlated with the proportion of the delayed regions (Figures 4B,D). Thus, the anti-adrenergic effect of muscarinic receptor stimulation on the amplitude of Ca^2+^ transients appears to be more pronounced with the increased level of detubulation of ventricular myocytes. Of note, the rate of Ca^2+^ transients decay did not correlate with the level of detubulation: in the presence of 100 nM Iso alone, the Pearson’s correlation coefficient was –0.27 and the slope of the regression curve was not different from 0 (*p* = 0.14). In the presence of 100 nM Iso plus 10 μM CCh, the relationship between the rate of Ca^2+^ transients decay and detubulation levels was fitted to the linear curve with the correlation coefficient –0.05 and the slope that was not different from 0 (*p* = 0.78).

The analysis of site-specific regulation of Ca^2+^ signaling revealed that CCh did not significantly affect the relationship between Ca^2+^ release from the coupled regions and the level of myocyte detubulation (Figure 4E). In contrast, the propagation of Ca^2+^ signal from sarcolemma, measured as the ratio of (d*F*/d*t*)_max_ recorded at the delayed regions to that recorded at the early regions, was significantly inhibited by the CCh (Figure 4F). It should be noted that the timing of Ca^2+^ release is affected not only by proximity to the sarcolemma but also by the functional properties of the sarcolemmal and SR Ca^2+^ channels (Zhou et al., 2009; Bryant et al., 2015). Therefore, we studied the effect of CCh on the timing of Ca^2+^ release by comparing the properties of Ca^2+^ transients recorded in the presence of Iso and Iso plus CCh in the same myocytes. First, we plotted the CCh-induced changes in the amplitude of Ca^2+^ transient vs. the fraction of delayed regions measured in the presence of Iso alone and confirmed that the inhibitory effect of CCh correlates with the level of myocyte detubulation (Figure 4G). Second, we found that CCh indeed produced a small (6%) but significant reduction of early regions and increased fraction of delayed regions by 8% (Figure 4H).

Taken together, these data suggest that downregulation of t-tubules in ventricular myocytes enhances the impact of muscarinic receptor stimulation on intracellular Ca^2+^ transient. This effect of CCh was associated with the inhibition of a small fraction of coupled SR Ca^2+^ release sites and weakened Ca^2+^ signal propagation from junctional release sites to uncoupled regions.

### CCh Does Not Affect the SR Ca^2+^ Content in Control and Formamide-Treated Ventricular Myocytes

**FIGURE 5:**
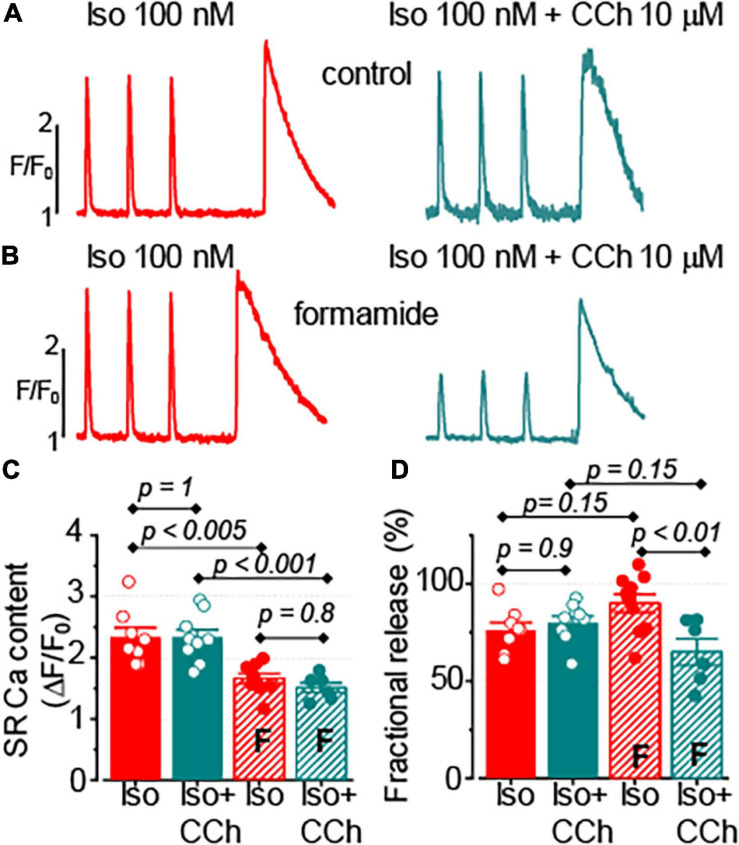
Muscarinic receptor stimulation does not affect the sarcoplasmic reticulum (SR) Ca^2+^ content in control and formamide-treated ventricular myocytes. **(A,B)** spatially averaged fluo-4FF fluorescence profiles illustrating field-stimulated (1 Hz) and caffeine-induced Ca^2+^ transients recorded in control **(A)** and formamide-treated **(B)** ventricular myocytes in the presence of 100 nM Iso alone, and in the presence of 100 nM Iso plus 10 μM CCh. Bar graphs with data points demonstrate the effect of CCh on the amplitude caffeine-induced Ca^2+^ transients **(C)** and fractional release **(D)** in control and formamide-treated (F) myocytes. The fractional release was calculated as the ratio of amplitudes of electrically-stimulated to caffeine-induced Ca^2+^ transients, respectively. Multiple pairwise comparisons were performed with Tukey’s test. The number of control myocytes studied was 14 for both Iso and Iso plus CCh groups. Number of formamide-treated myocytes studied was 10 for the Iso group and 6 for the Iso plus CCh group.

The SR Ca^2+^ release is known to be tightly regulated by the intra-SR Ca^2+^ levels (Györke et al., 2017). The effect of CCh on the SR Ca^2+^ content was assessed by studying caffeine-induced Ca^2+^ transients in myocytes loaded with fluo-4 FF Ca^2+^ sensitive dye. As illustrated in Figure 5, 10 μM CCh did not affect the SR Ca^2+^ content recorded in the presence of 100 nM Iso in control and in formamide-treated ventricular myocytes. Noticeably, the formamide treatment significantly reduced the SR Ca^2+^ load in myocytes challenged with either Iso alone or Iso plus CCh. Similar to the results obtained with fluo-4 dye, in experiments using fluo-4FF, CCh did not change the amplitude of field-stimulated at 1 Hz Ca^2+^ transients in control myocytes (1.77 ± 0.11 Δ*F*/*F*_0_, *n* = 14 in the presence of 100 nM Iso and 1.70 ± 0.12 Δ*F*/*F*_0_, *n* = 14 in the presence of 100 nM Iso plus 10 μM CCh, *p* = 0.66), but significantly inhibited it in formamide-treated myocytes (1.35 ± 0.12 Δ*F*/*F*_0_, *n* = 10 in the presence of 100 nM Iso and 0.92 ± 0.14 Δ*F*/*F*_0_, *n* = 6 in the presence of 100 nM Iso plus 10 μM CCh, *p* = 0.04). Furthermore, CCh significantly reduced fractional release in myocytes with reduced t-tubules density (Figure 5D). Analysis of time to peak of field stimulated Ca^2+^ transients revealed that the formamide treatment significantly increased this parameter for both Iso and Iso plus CCh groups indicating dyssynchrony of Ca^2+^ release activation consistent with the downregulation of t-tubules (Iso group: 33.75 ± 1.21 ms in control *vs.* 39.09 ± 1.72 ms in formamide-treated myocytes (*p* = 0.02); Iso plus CCh group: 33.43 ± 0.79 ms in control vs. 53.84 ± 4.52 ms in formamide-treated myocytes (*p* < 0.001) (Louch et al., 2010).

These results indicate that the inhibitory effect of CCh on the amplitude of Ca^2+^ transients cannot be explained by changes in the SR Ca^2+^ load. Rather, these point to the potential role of decreased Ca^2+^ influx and/or reduced Ca^2+^ sensitivity of the SR Ca^2+^ release channels as potential mechanisms mediating the effect of a muscarinic receptor agonist.

### Regulation of Sarcolemmal Ca^2+^ Channels by CCh

**FIGURE 6:**
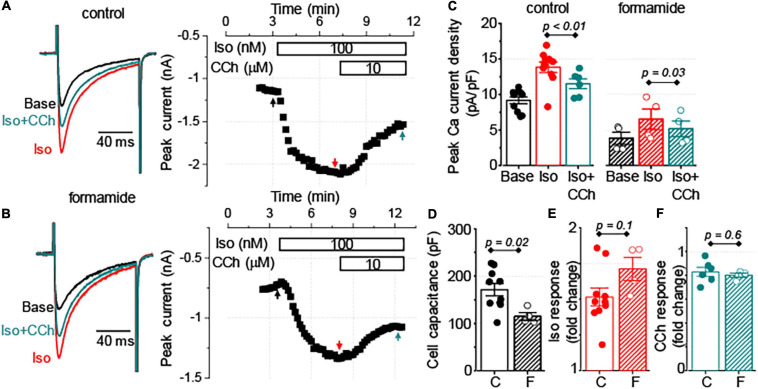
Similar regulation of sarcolemmal Ca^2+^ influx by muscarinic receptor stimulation in control and detubulated myocytes. **(A,B)** Representative traces of Ca^2+^ currents and corresponding time-courses of changes in the amplitude of Ca^2+^ currents observed at the baseline (Base), in response to application of 100 nM Iso alone followed by application of 100 nM Iso plus 10 μM CCh. Currents were recorded in control (**A**, *n* = 6–10) and formamide-treated (**B**, *n* = 4) ventricular myocytes. **(C)** Bar graphs with data points show the peak Ca^2+^ current density recorded at the baseline (Base), in the presence of Iso alone, and in the presence of Iso plus CCh in control **(C)** and formamide-treated **(F)** ventricular myocytes, respectively. Summary data along with individual data points are shown for myocyte capacitance **(D)**, peak Ca^2+^ current response to Iso, normalized to the baseline **(E)**, and peak Ca^2+^ current response to CCh, normalized to the peak Ca^2+^ current recorded in the presence of Iso **(F)**.

To test whether the signaling cross-talk between muscarinic and β-adrenergic receptor systems is different between t-tubular and peripheral sarcolemma, we compared the regulation of the L-type Ca^2+^ channel current (I_Ca_) was compared with Iso and CCh in control and formamide-treated ventricular myocytes. Formamide treatment reduced the cell capacitance by 33% and decreased the density of baseline Ca^2+^ current by 58% (Figures 6C,D). The application of 100 nM Iso to control and formamide-treated myocytes produced a similar 50–70% increase in the amplitude of peak Ca^2+^ current (Figures 6A–C,E). CCh (10 μM) inhibited this Iso-mediated increase in I_Ca_, on average, by 50% in both control and formamide-treated myocytes. However, when the effect of CCh was normalized to the amplitude of I_Ca_ recorded in the presence of Iso, it resulted in ∼20% inhibition of I_Ca_ in both groups (Figures 6A–C,F). These data indicate that signaling mediated by β-adrenergic and muscarinic receptors is similar for peripheral and t-tubular membranes, respectively.

### Formamide-Treatment Reduces Functional Reserve of Excitation–Contraction (EC) Coupling in Ventricular Myocytes

**FIGURE 7:**
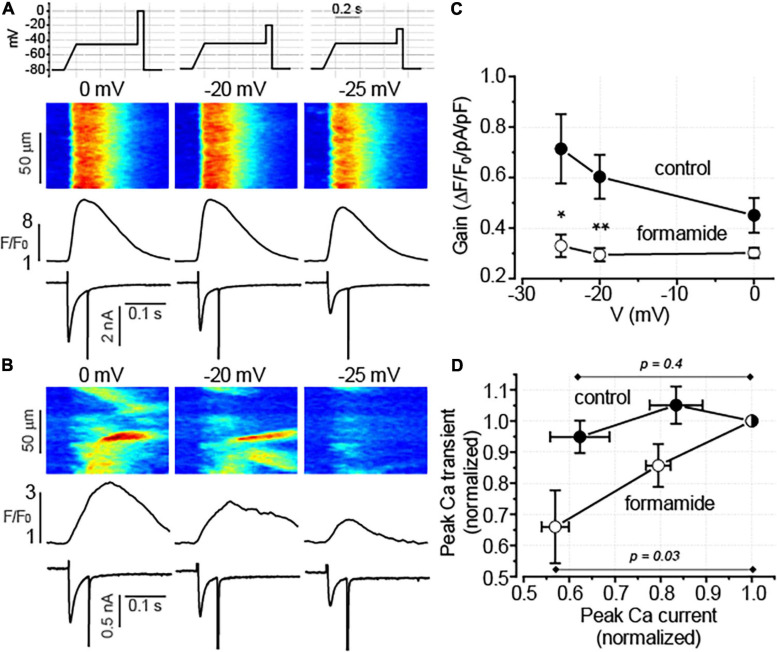
Detubulation reduces the fidelity of excitation-contraction (EC) coupling. **(A,B)** Representative line-scan images of fluo-4 fluorescence with the corresponding spatially averaged fluorescence profiles and I_Ca_ traces recorded in control **(A)** and formamide-treated **(B)** ventricular myocytes in a presence of 100 nM Iso. Depolarization levels used to evoke I_Ca_ are indicated above each line scan image. The upper panel in **A** illustrates the voltage protocol used to evoke Ca^2+^ currents. **(C)** The relationship between EC coupling gain and membrane voltage is shown for control (*n* = 5) and formamide-treated (*n* = 6) myocytes, respectively *p < 0.05; **p < 0.01 (control vs. formamide). **(D)** Voltage-dependent reduction in I_Ca_ decreased the amplitude of Ca^2+^ transients in formamide-treated but not in control myocytes, respectively. Data shown in panel C were transformed so that for each myocyte both peak Ca^2+^ transients and peak I_Ca_ were normalized to the corresponding values recorded at 0 mV.

The results demonstrate that in both control and formamide-treated ventricular myocytes, CCh similarly reduced I_Ca_ and did not affect the SR Ca^2+^ content. However, the inhibitory effect of CCh on Ca^2+^ transients in formamide-treated myocytes was observed in the context of significant reduction of both peak I_Ca_ density and the SR Ca^2+^ content. We hypothesize that the effect of CCh in formamide-treated myocytes is associated with the increased sensitivity of the SR Ca^2+^ release to changes in depolarization-induced Ca^2+^ influx. To test this hypothesis we studied how Ca^2+^ transients respond to changes in ICa amplitude in control and formamide-treated voltage-clamped myocytes. In these experiments, performed in the presence of 100 nM Iso, we sought to mimic the CCh-mediated inhibition of I_Ca_ by using different voltage protocols. As illustrated in Figure 7, in control ventricular myocytes changing the depolarization level from 0 to –25 mV resulted in 38 ± 6.5% (*n* = 5) reduction in the peak I_Ca_. This experimental protocol produced similar decrease in I_Ca_ amplitude (43 ± 3.0%, *n* = 6) in formamide-treated myocytes. However, in control ventricular myocytes, this decrease in I_Ca_ was not associated with the changes in Ca^2+^ transient (peak Ca^2+^ transients observed at –25 mV was 95 ± 5.2% of that recorded at 0 mV, *n* = 5, *p* = 0.4, paired *t*-test). In contrast, in formamide-treated myocytes, Ca^2+^ transients were significantly reduced by such a decrease in I_Ca_ (amplitude of Ca^2+^ transients observed at –25 mV was 66 ± 11% of that recorded at 0 mV, *n* = 6, *p* = 0.03, paired *t*-test) (Figure 7D). Accordingly, EC coupling gain measured at 0 mV in formamide-treated myocytes was 67 ± 4% of that measured in control myocytes (*p* = 0.05), whereas the gain recorded at –25 mV in formamide-treated myocytes was only 46 ± 6% of that measured in control myocytes (*p* = 0.02; Figure 6C). In aggregate, these data indicate the presence of a significant functional reserve (redundancy) of EC coupling in control myocytes during β-adrenergic receptor stimulation. Furthermore, the results show that the downregulation of t-tubules reduces EC coupling functional reserve thereby increasing the sensitivity of SR Ca^2+^ release to changes in the depolarization-induced Ca^2+^ influx.

### Inhibition of CaMKII With KN93 Mimics the Effect of CCh in Formamide-Treated Myocytes

The results that Ca^2+^ signal propagation is inhibited by CCh (Figure 4F) suggest that muscarinic receptor activation may affect the Ca^2+^ sensitivity of RyR2. Since no effect of CCh was found on the SR Ca^2+^ content, Ca^2+^ spark activity can serve as an indicator of Ca^2+^ sensitivity of RyR2s. Therefore, the frequency of Ca^2+^ sparks was examined following 1 Hz field-stimulation in formamide-treated myocytes that were challenged sequentially by Iso 100 nM alone and by Iso100 nM plus CCh 10 μM. Indeed, CCh significantly reduced Ca^2+^ spark frequency (Supplementary Figure 1) thereby supporting our hypothesis on the inhibition of RyR2 Ca^2+^ sensitivity by muscarinic receptor agonist in formamide-treated myocytes.

**FIGURE 8:**
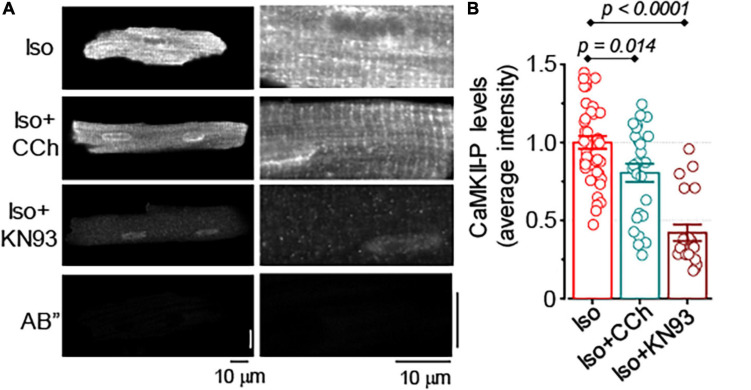
Muscarinic receptor stimulation reduces CaMKII activation in formamide-treated myocytes. **(A)** Representative images of formamide-treated myocytes immunostained for activated CaMKII (phospho T286). Right panels are the scaled-up parts of the corresponding left panel images. AB” images illustrate myocyte labeling with secondary antibody only. **(B)** Summary data along with individual data points illustrate average myocyte fluorescence observed in formamide-treated ventricular myocytes incubated with 100 nM Iso alone (*n* = 41), 100 nM Iso plus 10 μM CCh (*n* = 26), and 100 nM Iso and 2 μM KN93, a CaMKII inhibitor (*n* = 20). In all groups, myocytes were field-stimulated for 1 min at 2 Hz followed by 1 min at 1 Hz. Multiple pairwise comparisons were performed with Tukey’s test.

**FIGURE 9:**
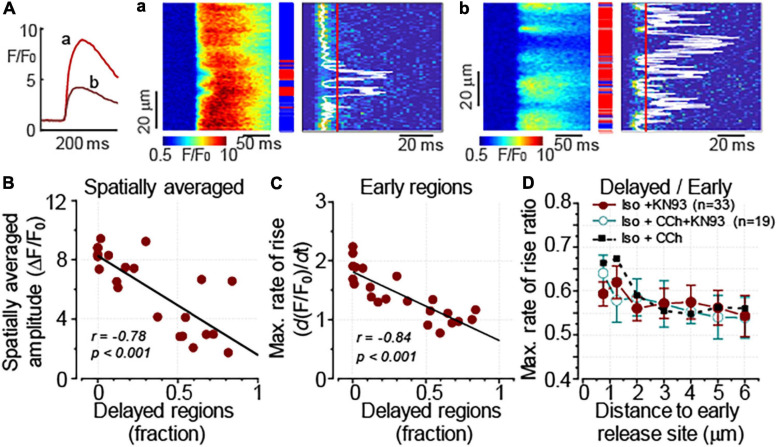
CaMKII inhibitor KN93 mimics the effect of CCh on Ca^2+^ transients in formamide-treated myocytes. Ca^2+^ transients were recorded in formamide-treated ventricular myocytes in the presence of 100 nM Iso and KN93 (2 μM). **(A)** Inset on the left shows spatially averaged fluorescence profiles recorded from the cell displaying moderate (a, corresponding to the line-scan image in panel a) and pronounced (b, corresponding to the line-scan image in panel b) levels of detubulation, respectively. **(A**,a,b**)** Linescan images of fluo-4 fluorescence (left) and corresponding images illustrating the rate of fluorescence change (right). The white line indicates the timing of (d*F/*d*t*)_max_, and the red line indicates the time point used to differentiate delayed regions (red rectangles). Early and intermediate regions are indicated by blue and gray rectangles, respectively. **(B)** The amplitude of Ca^2+^ transients is inversely correlated with the fraction of the delayed regions. **(C)** The relationship between (d*F/*d*t*)_max_ at the early Ca^2+^ release regions and the delayed region fraction recorded in the presence of Iso plus KN93 is shown along with a linear fit to the data. The slope of the regression line (–1.2 ± 0.2) was not significantly different (*F* test, *p* = 0.09) from that recorded in the presence of Iso plus CCh (shown in Figure 3E, –1.0 ± 0.2). **(D)** The propagation of Ca^2+^ from the sarcolemma was similarly affected by KN93 and CCh. The propagation was characterized by plotting the ratio of (d*F/*d*t*)_max_ recorded at the delayed regions to that measured at the early regions *vs.* the distance to the nearest early Ca^2+^ release site. Furthermore, CCh did not produce further changes in the (d*F/*d*t*)_max_ max ratio when added to the cells treated with KN93. Data obtained in the presence of Iso plus CCh (as in Figure 3F) are shown for comparison. **(B,C)** *r*, Pearson’s correlation coefficient; *p*, the probability that the slope of the regression curve is different from 0.

It has been previously reported that in control and failing ventricular myocytes CCh reduced CaMKII-dependent phosphorylation of RyR2, a post-translational modification that is strongly associated with the increased sensitivity of RyR2 to Ca^2+^ (Ho et al., 2016). In this study, the effect of CCh on CaMKII activity was examined in Iso-stimulated control and formamide-treated ventricular myocytes. In agreement with the results from field-stimulation experiments in control myocytes, in the presence of 100 nM Iso, CCh did not affect activation of CaMKII (Supplementary Figure 2). In contrast, in formamide-treated myocytes, in the presence of 100 nM Iso, CCh reduced CaMKII activation by 20% (Figure 8). For comparison, KN93 (2 μM), a well-characterized CaMKII inhibitor (Anderson et al., 1998), reduced CaMKII activity by 58% (Figure 8). Next, the effect of KN-93 was studied on the properties of Ca^2+^ release in formamide-treated myocytes in the presence of 100 nM Iso. As demonstrated in Figure 9, KN93 mimicked the effect of CCh on Ca^2+^ transients in formamide-treated myocytes. First, following KN93 treatment Ca^2+^ transient amplitude showed inverse correlation with the levels of detubulation, as in the case of CCh (Figures 9A,B). Second, in the presence of KN93 propagation of Ca^2+^ from coupled to delayed regions, as evidenced by normalized (d*F*/d*t*)_max_, was indistinguishable from that recorded in the presence of CCh (Figure 9D). In addition, the slope of the regression curve between (d*F*/d*t*)_max_ at early regions and delayed regions fraction was similar in myocytes treated with Iso plus KN93 and Iso plus CCh (Figures 4E, 9B). To test whether CCh produces any effects on Ca^2+^ propagation inside the cell in the presence of KN93, the (d*F*/d*t*)_max_ ratio was examined in myocytes treated with Iso plus CCh plus KN93. As shown in Figure 9D, the (d*F*/d*t*)_max_ ratio was not significantly affected by CCh, suggesting that CCh had no effects on Ca^2+^ propagation under these conditions.

Overall, these results suggest that the inhibitory effect of muscarinic receptor stimulation on Ca^2+^ transients observed in myocytes with downregulated t-tubules can be attributed, at least partially, to the reduced levels of CaMKII-dependent phosphorylation of RyR2.

## Discussion

A well-organized t-tubule system is critical for maintaining the robust excitation–contraction (EC) coupling in ventricular myocytes, whereas disorganization and downregulation of t-tubules have been consistently associated with the defective SR Ca^2+^ release in cardiac disease (Brette and Orchard, 2003; Louch et al., 2010; Guo et al., 2013; Poláková and Sobie, 2013). In this study, the mechanisms underlying the regulation of intracellular Ca^2+^ handling by muscarinic receptors in ventricular myocytes were addressed in the context of acutely disrupted t-tubules. Our main finding is that the downregulation of t-tubules amplified the inhibitory effect of muscarinic receptor agonist CCh on cytosolic Ca^2+^ transients. This effect is attributed to the increased dependency of SR Ca^2+^ release on CCh-mediated inhibition of both the L-type Ca^2+^ channel and RyR2.

### Muscarinic Receptor Signaling at the Surface Sarcolemma vs. T-Tubular Domain

In ventricular myocytes, the negative ionotropic effect associated with the stimulation of muscarinic receptors is predominantly attributed to the inhibition of β-adrenergic receptor–mediated stimulatory effects on Ca^2+^ transients and myocyte shortening (Hartzell, 1988; Endoh, 1999; Harvey and Belevych, 2003). Accordingly, we found that inhibitory effects of CCh on Ca^2+^ transients occurred only during β-adrenergic receptor stimulation with Iso (Figure 1). In this study, CCh at 10 μM was used to ensure maximal activation of muscarinic receptor–mediated functional responses (Vandecasteele et al., 1999). Growing evidence suggests that t-tubules have an important role not only in establishing and regulating compartmentalized Ca^2+^ and Na^+^ signaling (Bers, 2001; Brette and Orchard, 2003; Jones et al., 2018), but also for the local regulation of G-protein coupled receptor-mediated signaling, including signaling mediated by β-adrenergic receptors (Gorelik et al., 2013). To test the functional significance of muscarinic receptor signaling localized to t-tubular microdomains, we used a well characterized approach to acutely disrupt t-tubules with formamide treatment (Brette et al., 2002). In our experiments, the formamide treatment significantly reduced t-tubule density (Figures 2A–C) and enhanced the CCh-mediated inhibitory effect on the amplitude of Ca^2+^ transients (Figure 2G). We used the timing of maximal rate of increase of fluo-4 fluorescence as an index of proximity between the SR Ca^2+^ release sites and t-tubule sarcolemma (Dries et al., 2013, 2016; Belevych et al., 2017) to study the relationship between Ca^2+^ transient and detubulation level for each myocyte. This analysis showed that the amplitude of Ca^2+^ transients recorded in the presence of 100 nM Iso alone was not significantly affected by the extent of detubulation (Figure 4C). In contrast, the inhibitory effect of CCh was directly proportional to the degree of t-tubule downregulation (Figures 4D,G).

To explain this effect, we considered the possibility that β-adrenergic receptor and/or muscarinic receptor-mediated signaling is different at the surface sarcolemma vs. t-tubular domain. Indeed, Brette et al. (2004) showed different sensitivity of I_Ca_ to Iso in control and detubulated rat ventricular myocytes. Nikolaev et al. (2010) attributed the difference in β-adrenergic receptor-mediating signaling in surface vs. t-tubular membrane domain to the preferential localization of β2-adrenergic receptor to the t-tubular membrane of rat and mouse ventricular myocytes. Kashihara et al. (2014) reported preferential inhibition of t-tubular vs. surface sarcolemma Ca^2+^ channels by signaling mediated *via* β2-adrenergic and muscarinic receptors in mouse model of heart failure. However, in our experiment we did not find a significant difference in the sensitivity of I_Ca_ to Iso between the control and detubulated myocytes (Figure 6E), suggesting that in mouse ventricular myocytes β2-adrenergic receptor do not significantly contribute to the stimulatory effect of Iso. Importantly, no difference was observed in the response of I_Ca_ to CCh in control and detubulated ventricular myocytes (Figure 6F). Therefore, our data indicate that the molecular crosstalk between β-adrenergic and muscarinic receptor signaling is similar in t-tubular and surface membrane domains, at least at the level of L-type Ca^2+^ channel regulation.

### The Role of Excitation–Contraction-Coupling Reserve in Muscarinic Receptor Regulation of Ca^2+^ Transients

In ventricular myocytes, t-tubules support the spatial alignment of the L-type Ca^2+^ channels and RyR2, thus permitting the high fidelity coupling between the membrane depolarization and SR Ca^2+^ release (Franzini-Armstrong et al., 1999; Bers, 2001; Brette and Orchard, 2003). Downregulation of t-tubules drastically reduces depolarization-induced Ca^2+^ influx, significantly increases the fraction of RyR2s uncoupled from the L-type Ca^2+^ channels, and thereby, increases the contribution of regenerative Ca^2+^ release to the global Ca^2+^ transient (Brette et al., 2004). In the current experiments, formamide treatment was associated with a 50% decrease in I_Ca_ density in the presence of 100 nM Iso (Figure 6). Remarkably, despite these changes, the amplitude of Ca^2+^ transients was not affected by detubulation (Figure 4C). These results demonstrate the presence of a significant functional reserve of EC coupling in ventricular myocytes during β-adrenergic receptor stimulation. As it has been noticed in earlier studies, the linear relationship between sarcolemmal Ca^2+^ influx and SR Ca^2+^ release observed at baseline becomes flattened during β-adrenergic stimulation (Hussain and Orchard, 1997; Song et al., 2001). In other words, the increase in I_Ca_ amplitude beyond a certain level becomes redundant as it does not produce a further increase in the amplitude of Ca^2+^ transients. Accordingly, our experiments indicate that the presence of 100 nM Iso voltage-dependent reduction of I_Ca_ peak by 40% did not significantly change the amplitude of Ca^2+^ transients in control ventricular myocytes (Figure 7). In myocytes with downregulated t-tubules density, EC coupling reserve is significantly diminished and the SR Ca^2+^ release becomes more sensitive to changes in I_Ca_ density as demonstrated in experiments illustrated in Figure 7. Therefore, a similar reduction of I_Ca_ in control and detubulated ventricular myocytes induced by CCh (Figure 6F) may have a more pronounced effect on the Ca^2+^ transients in formamide-treated than in control myocytes.

### Regulation of Coupled and Uncoupled Ca^2+^ Release Sites by Muscarinic Receptor Stimulation

Analysis of time-dependent changes in fluorescence response to field stimulation (Figures 2D–F) allowed us to assess the effect of CCh on Ca^2+^ release sites functionally coupled and uncoupled from the t-tubules. The initial experiments in formamide-treated myocytes indicated that muscarinic receptor stimulation did not affect the properties of coupled Ca^2+^ release sites (Figure 4E). Further experiments in which we studied Ca^2+^ transients in the presence of Iso and Iso plus CCh in the same myocytes revealed that CCh reduced the fraction of coupled Ca^2+^ release sites by only 6% (Figure 4H). These results suggest that in the presence of Iso at 100 nM, a concentration producing a maximal stimulatory effect on Ca^2+^ transients (Figures 1, 2), muscarinic receptor stimulation has rather limited effects on regulation of adjacent to sarcolemma Ca^2+^ release sites. In contrast, the CCh significantly inhibited Ca^2+^ release at the uncoupled Ca^2+^ release sites (Figure 4F). This effect became evident with an apparent increase in the distance between coupled and uncoupled Ca^2+^ release sites, thus suggesting that CCh suppressed propagation of the Ca^2+^ signal from sarcolemma inside the cell. Propagation of Ca^2+^ under these conditions strongly relies on regenerative Ca^2+^ release at the uncoupled sites. This process is regulated by Ca^2+^ concentration inside the SR and by the sensitivity of RyR2 to cytosolic Ca^2+^ which is determined by both SR Ca^2+^ content and post-translational modifications of the protein (Brette et al., 2005; Venetucci et al., 2007; Belevych et al., 2013). In our experiments, CCh did not affect the SR Ca^2+^ content recorded in paced control and formamide-treated myocytes (Figure 5), suggesting a potential contribution of post-translational modifications of RyR2s in the CCh effect. β-Adrenergic receptor stimulation in ventricular myocytes has been associated with increased phosphorylation of RyR2 at Ser2808 and Ser2814 sites (Wehrens et al., 2005; Belevych et al., 2013) and oxidation-dependent intersubunit cross-linking (Bovo et al., 2012; Nikolaienko et al., 2018). All of these post-translational modifications result in the stimulation of RyR2 activity. We previously showed that CCh reduces phosphorylation at Ser2814, a CaMKII-dependent phosphorylation site (Ho et al., 2016). This effect was attributed to muscarinic receptor-mediated suppression of reactive oxygen species (ROS) generation and inhibition of downstream ROS signaling including activation of CaMKII. In this study, we showed that CCh inhibits activation of CaMKII in formamide-treated myocytes response to field stimulation in the presence of 100 nM Iso (Figure 8). Furthermore, myocyte pretreatment with KN93, a well-characterized inhibitor of CaMKII (Anderson et al., 1998), completely recapitulated the effect of CCh on Ca^2+^ transients in formamide-treated ventricular myocytes (Figure 9). Taken together, these data suggest that the inhibitory effect of CCh on the amplitude of Ca^2+^ transients in ventricular myocytes with disrupted t-tubules critically depends on inhibition of CaMKII-dependent phosphorylation of uncoupled RyR2 clusters.

CaMKII-dependent phosphorylation of RyR2 has been associated mostly with pathological remodeling and manifested as the increased myocyte propensity to arrhythmogenic Ca^2+^ waves (Anderson et al., 2011; Belevych et al., 2013; Mustroph et al., 2017). The previous studies showed that arrhythmogenesis in heart failure myocytes was attributable to CaMKII-dependent facilitation of coupled Ca^2+^ release site activity (Belevych et al., 2017). CaMKII-dependent activation of non-coupled Ca^2+^ release sites was also found important for Ca-dependent arrhythmogenesis in heart failure (Dries et al., 2018). The results of the present study suggest that CaMKII-dependent phosphorylation of uncoupled RyR2 may have physiological significance as the mechanism underlying EC coupling reserve.

### Limitations

Acute disruption of t-tubule organization in ventricular myocytes by formamide has been an invaluable tool for the research of the functional role of t-tubule domain and domain-related proteins (Brette et al., 2002, 2004, 2005; Despa et al., 2003; Chase and Orchard, 2011; Bryant et al., 2018). Furthermore, formamide-induced reduction of t-tubule density may resemble changes in the t-tubule system observed at the advanced stages of heart failure (Guo et al., 2013). However, in chronic cardiac diseases, such as heart failure, remodeling of the t-tubule system has been associated not only with reduced density and organization level but with changes in domain-specific protein expression and activity (Dries et al., 2013, 2016; Belevych et al., 2017; Jones et al., 2018). These changes may not be reproduced by acute detubulation protocol. Therefore, caution should be used when extrapolating these findings to disease settings.

## Conclusion

Heart failure has been associated with the diminished contribution of the parasympathetic nervous system in the regulation of cardiac function (Gold et al., 2016; van Bilsen et al., 2017). Although muscarinic receptors expressed in ventricular myocytes are known to play a significant role in mediating the effects of the parasympathetic nerve system, how heart failure affects muscarinic receptor-mediated signaling is not well defined (Fernandez and Canty, 2015). In this study, we described a novel t-tubule-dependent mechanism of cholinergic regulation of intracellular Ca^2+^ handling in ventricular myocytes that may be of importance in cardiac diseases, such as heart failure, which is associated with downregulated t-tubules (Brette and Orchard, 2003; Louch et al., 2010; Guo et al., 2013). The present study indicates that therapies involving parasympathetic augmentation (Hanna et al., 2018) may be associated with an unwanted muscarinic receptor-mediated inhibition of mechanical function in failing hearts.

## Data Availability Statement

The original contributions presented in the study are included in the article/Supplementary Material, further inquiries can be directed to the corresponding author.

## Ethics Statement

The animal study was reviewed and approved by The Ohio State University Institutional Animal Care and Use Committee.

## Author Contributions

AB, DT, and SG planned the research and wrote the manuscript. AB and VB designed and performed the experiments and analyzed the experimental data. All authors contributed to the article and approved the submitted version.

## Conflict of Interest

The authors declare that the research was conducted in the absence of any commercial or financial relationships that could be construed as a potential conflict of interest.

## Publisher’s Note

All claims expressed in this article are solely those of the authors and do not necessarily represent those of their affiliated organizations, or those of the publisher, the editors and the reviewers. Any product that may be evaluated in this article, or claim that may be made by its manufacturer, is not guaranteed or endorsed by the publisher.

## Footnotes

1
https://imagej.nih.gov/ij/

2
https://www.r-project.org
