# Supplementary material for: Acute Detubulation of Ventricular Myocytes Amplifies the Inhibitory Effect of Cholinergic Agonist on Intracellular Ca2+ Transients
Source: Front Physiol. 2021 Aug 26;12:725798. doi: 10.3389/fphys.2021.725798 (PMC8427700; doi:10.3389/fphys.2021.725798)
Supplement: Supplementary file 1 [file Data_Sheet_1.pdf]

Acute Detubulation of Ventricular Myocytes Amplifies the Inhibitory Effect of Cholinergic Agonist on Intracellular Ca^{2+} Transients.

Andriy E. Belevych, Vladimir Bogdanov, Dmitry A. Terentyev, Sandor Gyorke.

Supplementary Material

Supplemental Table 1. Properties of Ca^{2+} transients recorded in control ventricular myocytes electrically stimulated at 1 Hz.

	Iso alone		Iso + CCh 10 μM	
	Amplitude	Decay rate	Amplitude	Decay rate
Baseline (Iso 0 nM)	4.56±0.26 (26)	4.79±0.26 (26)	5.29±0.73 (13)	4.69±0.38 (13)
Iso 3 nM	7.20±0.38**## (19)	6.87±0.34**## (18)	4.96±0.48 (12)	4.89±0.53 (12)
Iso 10 nM	6.47±0.45**## (13)	6.05±0.62## (12)	4.74±0.35 (13)	4.23±0.21 (13)
Iso 30 nM	8.26±0.36** (16)	8.76±0.33** (16)	7.39±0.26* (13)	7.77±0.44** (13)
Iso 100 nM	6.97±0.49** (13)	10.61±0.84**(12)	7.84±0.53** (14)	10.00±0.66** (14)

Numbers in parenthesis indicate the number of myocytes studied. *, p<0.05, **, p<0.01 *vs.* baseline (Tuckey's test); #, p<0.05, ##, p<0.01 *vs.* CCh (Student's t-test)

Supplemental Table 2. Properties of Ca^{2+} transients recorded in formamide-treated ventricular myocytes electrically stimulated at 1 Hz**.**

	Iso alone		Iso + CCh 10 μM	
	Amplitude	Decay rate	Amplitude	Decay rate
Baseline (Iso 0 nM)	1.40±0.19 (15)	2.56±0.26 (15)		
Iso 3 nM	3.53±0.40**## (25)	4.90±0.38**## (25)	2.23±0.17 (27)	3.55±0.20 (27)
Iso 10 nM	3.82±0.27**## (17)	7.85±0.52**## (17)	2.64±0.33 (15)	4.53±0.37 (15)
Iso 30 nM	7.62±0.32**## (12)	9.92±0.45**## (12)	5.36±0.41 (18)	7.48±0.42 (18)
Iso 100 nM	6.87±0.34** (32)	11.15±0.38**## (32)	5.92±0.34 (39)	9.50±0.40 (39)

Numbers in parenthesis indicate the number of myocytes studied. **, p<0.01 *vs.* baseline (Tuckey's test); ##, p<0.01 *vs.* CCh (Student's t-test)

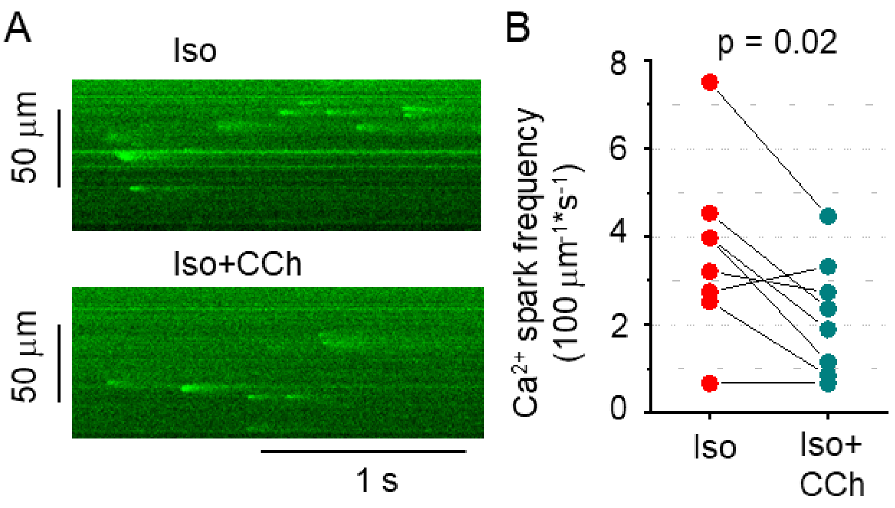

Supplemental Figure 1. CCh reduces Ca^{2+} spark frequency in formamide-treated myocytes. **(A)** Linescan images of fluo-4 fluorescence were recorded following field-stimulation at 1 Hz in formamide-treated myocytes in the presence of 100 nM Iso and in the presence of 100 nM Iso plus 10 µM CCh (n=8). **(B)** Summary data illustrating the effect of CCh on the frequency of Ca^{2+} sparks.

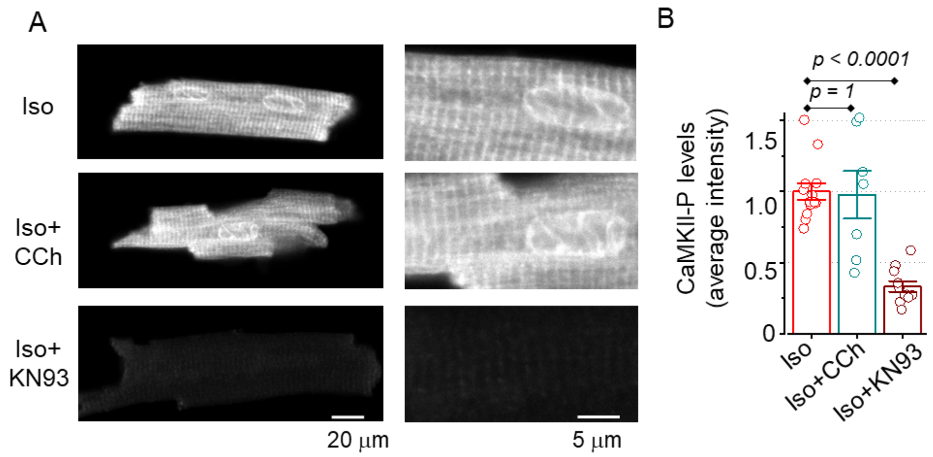

Supplemental Figure 2. Muscarinic receptor stimulation does not change CaMKII activation in control myocytes. **(A)** Representative images of control myocytes immunostained for activated CaMKII (phospho T286). Right panels are the scaled up parts of the corresponding left panel images. **(B)** Summary data along with individual data points illustrate average myocyte fluorescence observed in from control ventricular myocytes incubated with 100 nM Iso alone (n=13), 100 nM Iso plus 10 μM CCh (n=7), and 100 nM Iso and 2 μM KN93, a CaMKII inhibitor (n=11). In all groups myocytes were field-stimulated for 1 min at 2 Hz followed by 1 min at 1 Hz. Multiple pairwise comparisons were performed with Tukey's test.
